# Recurrent Hypokalemic Paralysis as the Initial Presentation of Primary Sjögren's Syndrome

**DOI:** 10.7759/cureus.115097

**Published:** 2026-08-24

**Authors:** Noor Ehsan, Shahbaz Mahmood, Usman Amjad, Ali Abbas Zahid, Muhammad Hassan Nawaz

**Affiliations:** 1 Internal Medicine, Gulab Devi Teaching Hospital, Lahore, PAK; 2 Family Medicine, Gulab Devi Teaching Hospital, Lahore, PAK; 3 Internal Medicine, Jinnah Hospital, Allama Iqbal Medical College, Lahore, PAK

**Keywords:** distal renal tubular acidosis, hypokalemia, hypokalemic periodic paralysis, normal anion gap metabolic acidosis, primary sjögren's syndrome

## Abstract

Distal renal tubular acidosis (dRTA) is associated with autoimmune disorders, particularly Sjögren's syndrome. Hypokalemic muscle weakness due to dRTA is an uncommon presentation of Sjögren's syndrome and may mimic primary hypokalemic periodic paralysis, resulting in a diagnostic delay. We present the case of a 35-year-old woman who reported recurrent limb weakness in the presence of hypokalemia (serum potassium: 2.7 mmol/L). She had previously been diagnosed with primary hypokalemic periodic paralysis, but this diagnosis was challenged as she was found to have normal anion gap metabolic acidosis and an alkaline urine pH with significant potassium wasting. The patient reported the presence of subtle sicca symptoms only on direct questioning. Further workup revealed positive serology for Sjögren's syndrome. She was managed with potassium and sodium bicarbonate replacement and given oral steroids and azathioprine for long-term management. Following correction of electrolyte abnormalities, the patient's muscle strength improved significantly, and she regained functional mobility. Her metabolic acidosis also resolved, and she experienced no further episodes of weakness or electrolyte imbalance during 12 months of follow-up. Primary Sjögren's syndrome-associated distal RTA should be considered in patients presenting with recurrent hypokalemic paralysis, particularly in the presence of normal anion gap metabolic acidosis and alkaline urine pH. Early treatment prevents recurrent paralysis and long-term renal complications.

## Introduction

Renal tubular acidosis (RTA) is an uncommon and often underdiagnosed condition [1]. Distal RTA (dRTA) may be primary or secondary to an autoimmune disease process, most commonly Sjögren's syndrome [2]. The prevalence of primary dRTA is <1:100,000, and secondary dRTA is approximately 10 times that of primary dRTA; nevertheless, dRTA remains a rare disorder [2]. It is characterized by an inability of the distal renal tubules to secrete hydrogen ions and reabsorb potassium ions. This phenomenon leads to the development of hypokalemia and normal anion gap metabolic acidosis [3]. A patient with dRTA may present with symptoms related to hypokalemia, such as weakness [4]. Based on this presentation, some of these patients may be mistakenly diagnosed with primary hypokalemic periodic paralysis [5].

Sjögren's syndrome is most commonly characterized by dryness of the eyes and oral cavity. Renal involvement is seen in about five percent of the cases, with tubulointerstitial nephritis (TIN) sometimes presenting with dRTA [6]. dRTA may have features ranging from recurrent renal stones to limb weakness [7]. While it may be a common presentation of dRTA, recurrent limb weakness as the sole presenting feature of Sjögren's syndrome is uncommon, with less than five percent of patients presenting this way [8].

We present the case of a 35-year-old female with recurrent episodes of limb weakness associated with hypokalemia. She had previously been diagnosed with primary hypokalemic periodic paralysis but was later found to have dRTA in the presence of Sjögren's syndrome.

## Case presentation

A 35-year-old female presented to the Medical Emergency Department with the complaint of progressively worsening limb weakness. Initially, the weakness was limited to the proximal muscles of both upper and lower extremities, but gradually it progressed to involve the distal muscle groups. Her weakness had rapidly worsened in the week preceding her presentation, leading to a complete inability to stand or walk. There was no history of bowel or bladder disturbance, sensory symptoms, or cranial nerve involvement. She reported no history of diarrhea, vomiting, diuretic use, laxative use, or any family history of periodic paralysis. She had had recurrent episodes of limb weakness for two years, followed by a severe episode six months before her current presentation. She was diagnosed with hypokalemic periodic paralysis based on her low serum potassium levels. She had been on long-term oral potassium replacement. However, her weakness persisted and even worsened over time. On further questioning, she reported long-standing fatigue, a gritty sensation in her eyes, dryness of the mouth, and dental caries. She did not have any history of joint pains, morning stiffness, malar rash, photosensitivity, or oral ulcers.

On examination, the patient was conscious, alert, and hemodynamically stable. Neurological examination revealed flaccid quadriparesis involving all four limbs with power 2/5 in the proximal and 3/5 in the distal muscle groups. Deep tendon reflexes were diminished (+1), and she had flexor plantar responses bilaterally. Sensory examination was intact for all modalities. Cranial nerve examination was unremarkable, and there were no signs of meningeal irritation or cerebellar involvement.

**Figure 1:**
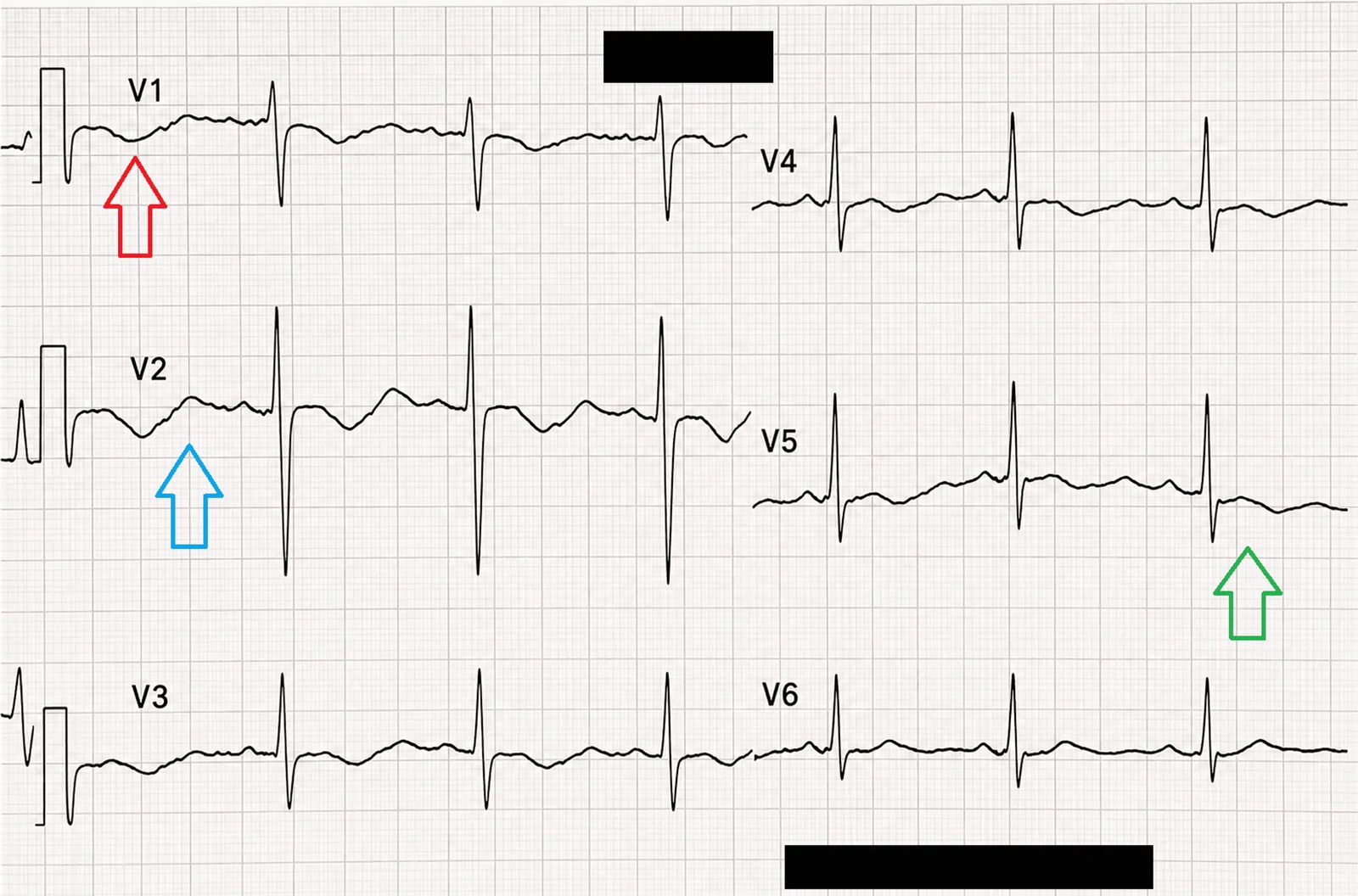
Electrocardiogram Electrocardiogram with findings suggestive of hypokalemia in chest leads. The red arrow points to T wave inversion, the blue arrow points to the U wave, and the green arrow shows a flattened T wave. The QT interval corrected for the patient's heart rate was calculated to be 358 ms (normal range: 360-460 ms). Patient identifying information has been digitally concealed using black bars.

Initial laboratory investigations revealed severe hypokalemia, elevated serum chloride, and low serum bicarbonate levels. Arterial blood gas analysis showed metabolic acidosis in the presence of a normal serum anion gap. Her urine pH was inappropriately alkaline in the presence of systemic acidosis, and her renal function tests were normal (Table 1). No proteinuria was detected, and urinalysis was unremarkable. Her 24-hour urine potassium level was 60 mmol/24 hours, which was inappropriately high given her low serum potassium levels. She had a positive urine anion gap (Table 2). Her electrocardiogram showed sinus rhythm with inversion and flattening of T-waves along with U-waves in the chest leads (Figure 1). These findings were consistent with normal anion gap metabolic acidosis and dRTA. Ultrasound of the kidneys was normal and showed no evidence of nephrolithiasis or nephrocalcinosis. Autoimmune workup revealed positive titres for antinuclear antibody (ANA), anti-Sjögren's syndrome-related antigen A (SSA/Ro) antibody, and anti-Sjögren's syndrome-related antigen B (SSB/La) antibody, supporting the diagnosis of primary Sjögren's syndrome (Table 3). The rest of the extractable nuclear antigen (ENA) profile was negative. Ophthalmology was consulted, and the Schirmer test was performed (without a topical anesthetic), which showed less than 5 mm of wetting in the right eye and 6 mm in the left eye after five minutes. This was indicative of reduced tear production. Her 2016 American College of Rheumatology/European Alliance of Associations for Rheumatology (ACR/EULAR) classification criteria for primary Sjögren syndrome score was four [9]. Based on these investigations and the 2016 ACR/EULAR criteria [9], she was diagnosed with dRTA secondary to primary Sjögren's syndrome. 

**Table 1:** Laboratory investigations

Investigation	Patient Value	Normal Range
Serum potassium	2.7 mmol/L	3.5-5.5 mmol/L
Serum sodium	138 mmol/L	135-145 mmol/L
Serum chloride	115 mmol/L	95-105 mmol/L
Serum bicarbonate	14 mmol/L	18-22 mmol/L
Blood pH	7.32	7.35-7.45
Serum anion gap	9 mmol/L	8-16 mmol/L
Urine pH	6	4.5-8.0
Serum creatinine	0.8 mg/dL	0.1-1.0 mg/dL

**Table 2:** Laboratory investigations

Investigation	Patient Value	Normal Range
Urine anion gap	+50 mmol/L	-10 to +10 mmol/L
Spot urine sodium	40 mmol/L	>20 mmol/L
Spot urine potassium	50 mmol/L	15-40 mmol/L
Spot urine chloride	40 mmol/L	30-290 mmol/L

**Table 3:** Serological workup Anti-SSA: Anti-Sjögren's syndrome-related antigen A, Anti-SSB: Anti-Sjögren's syndrome-related antigen B

Investigation	Patient Value	Normal Range
Antinuclear antibody titre	1:5120	<1:80
Anti-SSA/Ro 60 kd antibody titre	2.1	Weak positive: >1.15-2.5 Strong positive: >2.5
Anti-SSA/Ro 52 kd antibody titre	2.7	Weak positive: >1.15-2.5 Strong positive: >2.5
Anti-SSB/La antibody titre	2.5	Weak positive: >1.15-2.5 Strong positive: >2.5

Other possible differential diagnoses, such as thyrotoxic periodic paralysis, Gitelman syndrome, Bartter syndrome, and hyperaldosteronism, were ruled out based on normal thyroid function tests; the presence of renal potassium wasting, hyperchloremic normal-anion-gap metabolic acidosis, impaired urinary acidification; and the lack of elevated blood pressure readings. Gastrointestinal losses were excluded based on the absence of diarrhea, vomiting, and laxative use, along with the presence of inappropriate urinary potassium excretion and a positive urine anion gap. Hereditary hypokalemic periodic paralysis was considered, but the presence of persistent renal potassium wasting and normal-anion-gap metabolic acidosis with impaired distal urinary acidification argued strongly against a primary channelopathy as the underlying cause. Surreptitious diuretic use was excluded based on her metabolic profile, which was inconsistent with diuretic-induced potassium wasting and hypokalemic metabolic alkalosis.

The patient was managed with intravenous potassium replacement initially, followed by oral potassium and sodium bicarbonate replacement. Gradual improvement in muscle strength was observed over the next few days, and the patient regained the ability to stand and walk. Given the significant systemic involvement of primary Sjögren's syndrome with distal RTA and recurrent hypokalemic paralysis, systemic immunosuppressive therapy was initiated, as per the EULAR guidelines [10]. She was given weight-based oral prednisone, tapered over a month. She was also started on azathioprine 50 mg once daily, later increased to 50 mg twice daily. Ocular lubricant drops were used to relieve eye symptoms. She was counseled regarding the need for long-term compliance with her medication. After being discharged, she has followed up with us in the outpatient department and has reported no further episodes of weakness during the past 12 months. Her hypokalemia and metabolic acidosis have also resolved, with recent biochemical testing mentioned in Table 4. 

**Table 4:** Laboratory investigations

Investigation	Patient Value	Normal Range
Serum potassium	3.6 mmol/L	3.5-5.5 mmol/L
Serum bicarbonate	20 mmol/L	18-22 mmol/L
Blood pH	7.37	7.35-7.45
Urine pH	5.5	4.5-8
Serum creatinine	0.9 mg/dL	0.1-1.0 mg/dL

## Discussion

dRTA, also known as type 1 renal tubular acidosis, is a complex entity characterized by an inability to acidify urine, a process that occurs in the collecting tubules and collecting ducts of nephrons [11]. The condition may be primary or secondary (acquired) [2]. The pathophysiology includes a net reduction in hydrogen ion secretion in the distal part of the nephron, which results in impaired bicarbonate regeneration [12]. This results in a normal anion gap metabolic acidosis, alkaline urine, and hypokalemia [13]. The impaired hydrogen ion secretion may be caused by decreased activity of H⁺-ATPase, H⁺/K⁺-ATPase (secretory defect), or increased permeability of the luminal membrane (gradient defect) [12]. In adults, dRTA is frequently associated with autoimmune diseases and medications, with primary Sjögren's syndrome being one of the most commonly reported autoimmune causes [11].

Sjögren's syndrome is a connective tissue disorder most commonly characterized by dryness of the eyes and mouth, collectively known as keratoconjunctivitis sicca. Salivary and tear-producing glands are the most common tissue targets in Sjögren's syndrome [9]. However, the disease can also affect other parts of the body, and sometimes, features of extraglandular involvement precede those of glandular damage. Extraglandular tissues that may be involved in Sjögren's syndrome include the kidneys, peripheral nervous system, lungs, skin, and joints. In some cases, the patients initially do not present with sicca symptoms, and the lack of classic symptoms may make it harder to diagnose the disease [14]. Untreated disease could lead to nephrolithiasis, nephrocalcinosis, chronic kidney disease, TIN, and glomerular disease [13].

Renal involvement in Sjögren's syndrome most commonly occurs in the form of TIN, which may manifest as dRTA [14]. Pathophysiology includes damage to the alpha intercalated cells in the collecting ducts and antibodies against carbonic anhydrase II [15,16]. Damage to the H⁺/K⁺-ATPase in the alpha intercalated cells leads to the development of hypokalemia, which can present with limb weakness [1]. This is what happened in our case, where the patient had recurrent attacks of lower limb weakness in the presence of hypokalemia. She was previously diagnosed with primary hypokalemic periodic paralysis. However, her bloodwork revealed the presence of normal anion gap metabolic acidosis, which challenged the diagnosis of primary hypokalemic periodic paralysis and prompted further workup for dRTA. The diagnosis was further supported by the presence of high urine pH (6.0) and potassium wasting on urine studies. Other important causes of hypokalemic weakness include gastrointestinal losses, Gitelman syndrome, Bartter syndrome, and primary hyperaldosteronism. However, they were not likely in our case, as our patient had metabolic acidosis and consistently normal blood pressure readings [17]. The diagnosis of dRTA was further supported by a urine pH of 6 and a positive urine anion gap, indicating impaired urinary ammonium excretion [18].

Muscle weakness due to hypokalemia secondary to dRTA is an uncommon presentation of Sjögren's syndrome, but such cases have been reported before. Meena et al. and Garza-Alpirez et al. reported similar cases in which hypokalemic paralysis was the presenting feature of Sjögren's syndrome-associated dRTA [19,20]. In such cases, renal symptoms can precede sicca symptoms by months to years [20]. Our patient reported the presence of oral dryness and eye dryness for six months prior to her presentation. Her sicca symptoms were milder, and they were overshadowed by the profound, episodic lower limb weakness, which she had been experiencing for two years. The combination of strongly positive ANA, anti-Ro/SSA, anti-La/SSB antibodies, and a positive Schirmer test established the diagnosis of primary Sjögren's syndrome and identified the underlying cause of the patient's dRTA. Our patient responded well to both electrolyte replacement therapy and azathioprine. She has remained symptom-free and metabolically stable since the initiation of treatment.

## Conclusions

Clinicians should maintain a high index of suspicion for dRTA in patients presenting with recurrent hypokalemic paralysis, particularly when accompanied by normal anion gap metabolic acidosis and an alkaline urine pH. In adult women with unexplained dRTA, Sjögren's syndrome should be investigated.
